# Airway Smooth Muscle Cell Mitochondria Damage and Mitophagy in COPD via ERK1/2 MAPK

**DOI:** 10.3390/ijms232213987

**Published:** 2022-11-12

**Authors:** Lei Fang, Ming Zhang, Junling Li, Liang Zhou, Michael Tamm, Michael Roth

**Affiliations:** 1 Pulmonary Cell Research, Department of Biomedicine, University of Basel, 4031 Basel, Switzerland; 2 Clinic of Respiratory Medicine, Department of Internal Medicine, University Hospital Basel, 4031 Basel, Switzerland; 3 Department of Respiratory and Critical Care Medicine, The Second Affiliated Hospital of Xi’an Jiaotong University, Xi’an 710049, China; 4 The First Dongguan Affiliated Hospital of Guangdong Medical University, Dongguan 523000, China

**Keywords:** COPD, airway smooth muscle cells, autophagy, mitophagy, ERK1/2, mitochondria fragmentation, cigarette smoke

## Abstract

Chronic obstructive pulmonary disease (COPD) is characterized by irreversible deterioration of the airway wall. Cigarette smoking is the major trigger, and in vitro studies showed that cigarette smoke extract (CSE) induced mitophagy in airway epithelial cells via oxidative stress, but this mechanism was not studied in airway smooth muscle cells (ASMCs). Primary ASMCs isolated from COPD patients or non-disease donors were investigated for CSE-induced remodeling and mitochondria structure. Proteins were assessed by Western blots for remodeling: collagen type-I, α-smooth muscle actin (α-SMA) and fibronectin; autophagy: beclin-1, protein62 (p62), light chain (LC)3A/B; mitochondria activity: mitochondrially encoded cytochrome c oxidase II & -IV (MTCO2, MTCO4), peroxisome proliferator activated receptor gamma coactivator 1α (PGC-1α); lysosomes: early endosome antigen 1, lysosome activated membrane protein 1; and cell signaling: extracellular signal regulated kinase (ERK1/2). Lysotracker and Mitotracker were used to monitor mitochondria morphology and organelle co-localization. Compared with controls, untreated COPD ASMCs showed lower collagen type-I and α-SMA expressions, but increased fibronectin levels. CSE further downregulated collagen type-I and α-SMA expression, but upregulated fibronectin. CSE decreased PGC-1α, MTCO2, and MTCO4, but increased beclin-1, p62, and LC3. CSE upregulated mitophagy and lysosomes activity via ERK1/2 phosphorylation. In vitro, cigarette smoke induced the deterioration of ASMCs, which might explain the tissue loss and structural remodeling in COPD bronchi. The results suggest that preventing exceeded mitophagy in ASMCs might present a novel therapeutic target for COPD.

## 1. Introduction

Mitochondria are the power station of all cells and therefore the loss of their number or activity, including exceeded mitophagy, will impair cell function and tissue homeostasis as it has been shown in vascular smooth muscle cells and cardiovascular diseases [1]. The balance between fission and fusion of mitochondria is essential for cell function, as they control the cell metabolism, regulate inflammation, or lead to regulated cell death (RCD) and being removed by mitophagy [2,3]. Mitochondria play a central role in health and disease, as well as in aging [4] and many lung disorders [5]. However, the impact of mitophagy in chronic inflammatory lung diseases, such as asthma and chronic obstructive pulmonary disease (COPD), has not been studied in much detail [6]. 

Airway smooth muscle cells (ASMCs) play a central role in the pathogenesis of chronic inflammatory lung diseases, including COPD, and are regarded as a driver of tissue remodeling [7]. Oxidative stress and mitochondrial dynamics have been shown to affect airway epithelial and smooth muscle cells homeostasis and repair, suggesting that the role of mitochondria has to be further investigated in chronic inflammatory lung diseases [8]. In ASMCs, oxidative stress increased the consumption of oxygen and resulted in inflammation-dependent protein unfolding [9]. However, compared to bronchial epithelial cells, the effect of cigarette smoke on ASMCs is not well known [10]. 

In bronchial epithelial cells, it has been shown that cigarette smoke activates the cell through toll-like receptor 4 (TLR4) and the simultaneous modification of mitochondria activity and the formation of radical oxygen species (ROS) [11]. Cigarette smoke extract (CSE) increased the expression of microRNA-21 (miR-21) and extracellular signal-regulated kinase 1/2 (ERK1/2) through activating TLR4 [11]. CSE-induced mitochondria fragmentation correlated with increased ROS production, while PINK1 (PTEN-induced putative kinase 1)–parkin pathway-regulated mitophagy led to cell senescence [12,13]. In ASMCs, the effect of IgE (immunoglobulin E) on mammalian target of rapamycin (mTOR) signaling and mitochondria dysfunction was reported in an asthma remodeling model [14]. IgE upregulated miR-21-5p, leading to increased expression of the mTOR, peroxisome proliferator activated receptor gamma (PPAR-γ), PPAR-γ coactivator 1α (PGC1-α), cyclooxygenase-2, and mitochondrial activity, leading to increased proliferation and remodeling [14]. In ASMCs, PGC-1α activated the parkin mitophagy pathway, which was dependent on mitochondria fusion and induced mitophagy [15]. However, the role of PPAR-γ in the regulation of ASMC proliferation is controversially reported and may depend on specific ligands and conditions [16,17]. In human cells and a COPD animal model, CSE increased COPD-related parameters, including collagen type-I and proliferation through activating ERK1/2 [18]. In another study, CSE exposure (1%, 48 h) tipped the balance between mitochondria fission and fusion, and thereby controlled ASMC proliferation [19]. 

This study investigated the effect of cigarette smoke extract (CSE) on primary human ASMCs obtained from COPD patients and non-diseased controls. The role of mitochondria damage and mitophagy was also investigated.

## 2. Results

### 2.1. Dysregulation of Mitochondria Drives ASMC Remodeling in COPD

**Figure 1:**
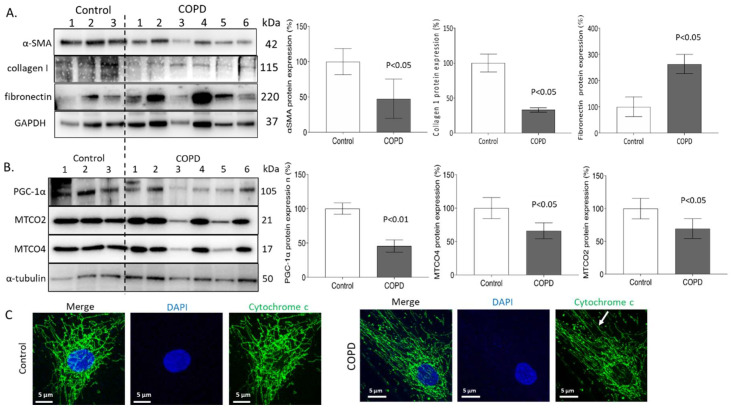
CSE modulates remodeling and induces mitochondria fragmentation in ASMCs. (**A**) Representative Western blots and image analysis of α-SMA, collagen type-I, and fibronectin protein expressions of ASMCs of control and COPD patients. GAPDH (glyceraldehyde 3-phosphate dehydrogenase) served as house-keeping protein; *p* < 0.05 was considered significant. (**B**) Representative Western blots and image analysis of MTCO2, MTCO4, and PGC-1α protein by ASMCs of control and COPD patients; tubulin was used as reference protein and *p* < 0.05 was considered as significant. Statistics were calculated by Student’s *t*-test. (**C**) Mitochondria morphology in ASMCs of control and COPD patients by confocal microscopy (60×, green: cytochrome C, blue: DAPI (4’,6-diamidin-2-phenylindol), white arrow indicates fragmented mitochondria).

Compared to cells derived from non-disease donors (*n* = 3), ASMCs derived from COPD patients (*n* = 6) showed lower expression of α-SMA (47.3%) and collagen type-I (32.9%), while the expression of fibronectin was significantly higher (262.9%) (Figure 1A). The concentration-dependent effect of CSE on fibronectin and collagen type-I are shown in Figure S1A. In addition, the expression of mitochondrial stimulating PGC-1α, and mitochondrial functional proteins mitochondrially encoded cytochrome c oxidase II & -IV (MTCO2, MTCO4) were all significantly decreased in primary ASMCs of COPD patients when compared with controls (45.6%, 69.4%, and 66.1%, respectively, Figure 1B). The concentration-dependent effects of CSE on MTCO2 and MTCO4 are shown in Figure S1A. As shown by immunofluorescence confocal microscopy, ASMCs of COPD patients have impaired mitochondria morphology, such as fragmentation, when compared with non-diseased ASMCs (Figure 1C).

### 2.2. ASMCs of COPD Patients Showed Increased Expression of Proteins Enhancing Autophagy, Lysosome Activity, and Mitochondrial Damage

**Figure 2:**
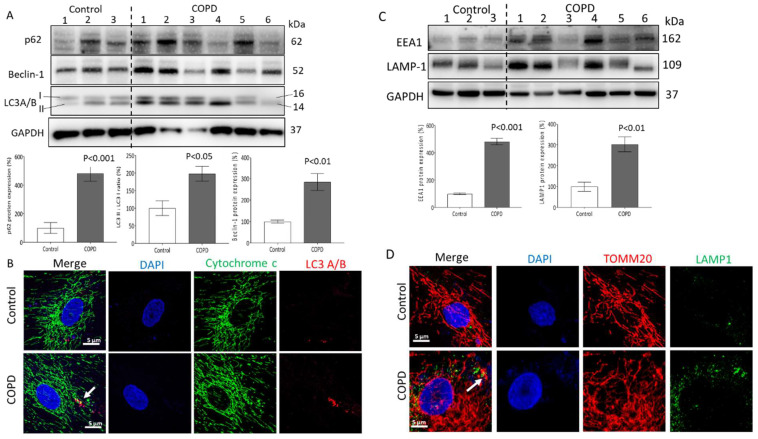
CSE modulates mitochondrial homeostasis by mitophagy and lysosome activity. (**A**) Representative Western blots and image analysis of p62, beclin-1, and LC3A/B protein of control and COPD ASMCs; GAPDH served as reference protein; *p* < 0.05 was considered significant. Statistics were calculated by Student’s *t*-test. (**B**) Representative confocal microscopy of co-localization mitochondria-LC3 (60×, green: cytochrome C, red: LC3A/B, blue: DAPI, white arrow shows co-localization). (**C**) Representative Western blots and image analysis of EEA1 and LAMP-1 protein of control and COPD ASMCs; GAPDH was used as reference protein, *p* < 0.05 was considered significant. Statistics were calculated by Student’s *t*-test. (**D**), Representative confocal microscopy of mitochondria–lysosome co-localization (60×, red: TOMM20, green: LAMP1, blue: DAPI, white arrow shows co-localization.

The expression of different autophagy markers in ASMCs obtained from COPD patients was significantly increased when compared to cells of controls. As shown by representative Western blots in Figure 2A, ASMCs of COPD patients showed a higher expression of p62 (481.8%) and Beclin-1 (285.3%), as well as an increased ratio of LC3A/B II to LC3A/B I (198.2%) when compared with the control. The latter was confirmed by immunofluorescence co-staining, with increased LC3A/B II in COPD cells, and its co-localization with cytochrome C, indicating increased mitophagy in ASMCs of COPD patients (Figure 2B). The concentration-dependent effect of CSE on the three proteins is shown in Figure S1B.

In Figure 2C, protein analysis by Western blot showed that the expression of early endosome antigen 1 (EEA1) and lysosomal-associated membrane protein 1 (LAMP-1) were both significantly increased in ASMCs of COPD patients (481.7% and 302.9%, respectively), when compared to control cells. The increased expression of LAMP-1 in ASMCs of COPD patients co-localized with damaged mitochondria is shown by co-staining (Figure 2D, white arrow). The concentration-dependent effect of CSE on the two proteins is shown in Figure S1B.

### 2.3. CSE-Induced ASMC Remodeling and Mitochondrial Damage

**Figure 3:**
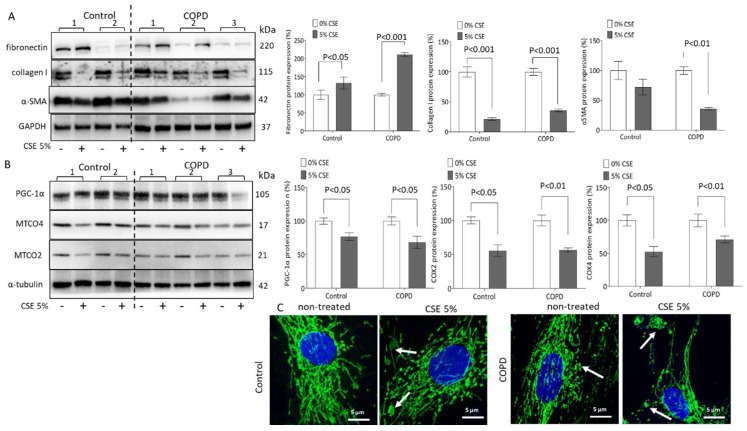
CSE modifies ASMC remodeling and reduces mitochondria activity. (**A**), Representative Western blots and image analysis of CSE-induced fibronectin, collagen type-I, and α-SMA protein changes. GAPDH served as a reference protein and *p* < 0.05 was considered significant. (**B**) Representative Western blot and image analysis of CSE-induced MTCO2, MTCO4, and PGC-1α protein expressions. α-Tubulin served as a reference protein and *p* < 0.05 was considered significant. Statistics were calculated by Mann–Whitney U-test. (**C**) Representative fluorescence microscopy of CSE-induced mitochondria morphology (60×, green: cytochrome C, blue: DAPI, white arrow indicates fragmented mitochondria or released cytochrome C).

Treatment with CSE (5%, 24 h) significantly increased fibronectin expression in both the control (132.1%) and ASMCs of COPD patients (210.9%) when compared to non-treated cells (Figure 3A). This effect was stronger in ASMCs of COPD patients compared to control cells. In contrast, CSE decreased the expression collagen type-I significantly in both the control (21.2%) and COPD (35.67%) cells (Figure 3A). The expression of α-SMA was also significantly decreased by CSE in ASMCs of COPD patients (35.7%, Figure 3A). 

In addition, CSE treatment decreased PGC-1α expression in both control (76.7%) and COPD (68.1%) cells (Figure 3B). Furthermore, CSE significantly downregulated the expression of mitochondrial proteins MTCO2 and MTCO4. The expression of MTCO2 was reduced by 50% after 24 h exposure to CSE in both cell types (Figure 3B). CSE reduced MTCO4 levels by 50% in control cells and by 30% in COPD cells (Figure 3B). 

As shown in Figure 3C, non-treated ASMCs of COPD patients presented with fragmented mitochondria (white arrow). Exposure to CSE increased the mitochondria fragmentation further in cells of COPD patients and induced fragmentation in control cells (Figure 3C). Color-dissected photographs of Figure 3C are shown in Figure S2.

### 2.4. CSE-Activated Mitophagy and Lysosome Activity in ASMCs

**Figure 4:**
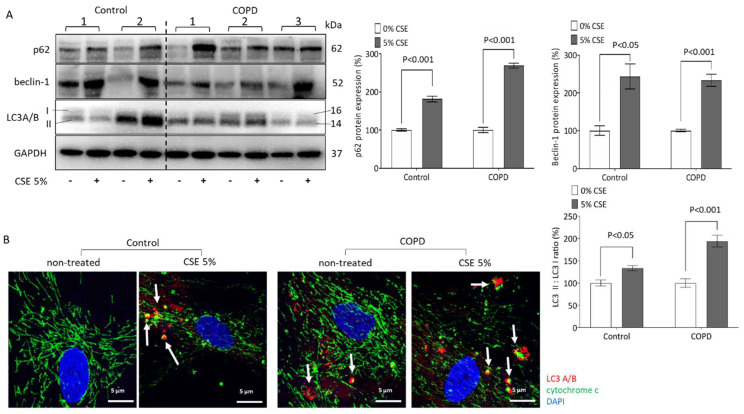
CSE induces autophagy in ASMCs. (**A**) Representative Western blots and image analysis of CSE-induced p62, beclin-1, and LC3A/B protein expression. GAPDH served as reference protein and *p* < 0.05 was considered significant. (**B**) Representative confocal microscopy on mitochondria-LC3 co-localization (white arrow) after CSE-stimulation (60×, green: cytochrome C, red: LC3A/B, blue: DAPI). Statistics were calculated by Mann–Whitney U-test.

In both cell types, COPD and control ASMCs, treatment with CSE over 24 h upregulated the expression of intracellular autophagy proteins significantly. The expression of p62 was upregulated by CSE to 181.3% in control cells, and to 268.9% in COPD cells, when compared with untreated cells in the same diagnostic group (Figure 4A). Interestingly, CSE doubled the level of beclin-1 in both control and COPD cells (Figure 4A). Furthermore, the ratio of LC3A/B II to LC3A/B I was significantly increased in both control and COPD cells; however, it had a stronger effect in COPD cells (control 133.4%, COPD 193.9%, Figure 4A).

Co-staining for LC3 and mitochondrial cytochrome C revealed that both were increased in CSE treated cells (Figure 4B). Furthermore, treatment with CSE caused the co-localization of both proteins, which is shown in Figure 4B by yellow fluorescence color. Color-dissected photographs of Figure 4B are shown in Figure S3.

**Figure 5:**
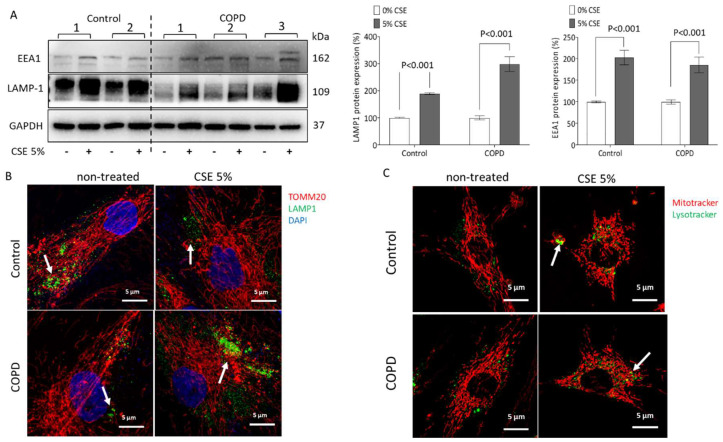
CSE induces lysosome activity in ASMCs. (**A**) Representative Western blots and image analysis of CSE-induced EEA1 and LAMP1 protein expression. GAPDH served as reference protein and *p* < 0.05 was considered significant. Statistics were calculated by Mann–Whitney U-test. (**B**) Representative confocal microscopy of mitochondria–lysosome co-localization after CSE-stimulation (60×, red: TOMM20, green: LAMP1, blue: DAPI). (**C**) Representative live cell imaging of mitochondria–lysosome co-localization (white arrow) after CSE-stimulation. (60×, red: Mitotracker, green: Lysotracker, white arrow indicates co-localization).

CSE treatment significantly upregulated the expression of the early endosome marker EEA1 in ASMCs of controls (202.7%) and COPD patients (185.9%) (Figure 5A). Furthermore, the expression of the late endosome marker LAMP-1 was significantly increased by CSE in both groups (control 189.2%, COPD 298.3%, Figure 5A). 

Co-staining for TOMM20 and LAMP-1 demonstrated that CSE significantly damaged the morphology of mitochondria and increased their fragmentation, and it also upregulated lysosome activity (Figure 5B). Color-dissected photographs of Figure 5B are shown in Figure S4. 

To verify the co-localization of damaged mitochondria and lysosomes after CSE stimulation, a live-cell confocal microscopy imaging experiment using Lysotracker and Mitotracker was performed as shown in Figure 5C (green/yellow staining). Color-dissected photographs of Figure 5C are shown in Figure S5.

### 2.5. CSE-Induced Mitophagy of ASMCs Is Mediated through the Activation of ERK1/2 Signaling

**Figure 6:**
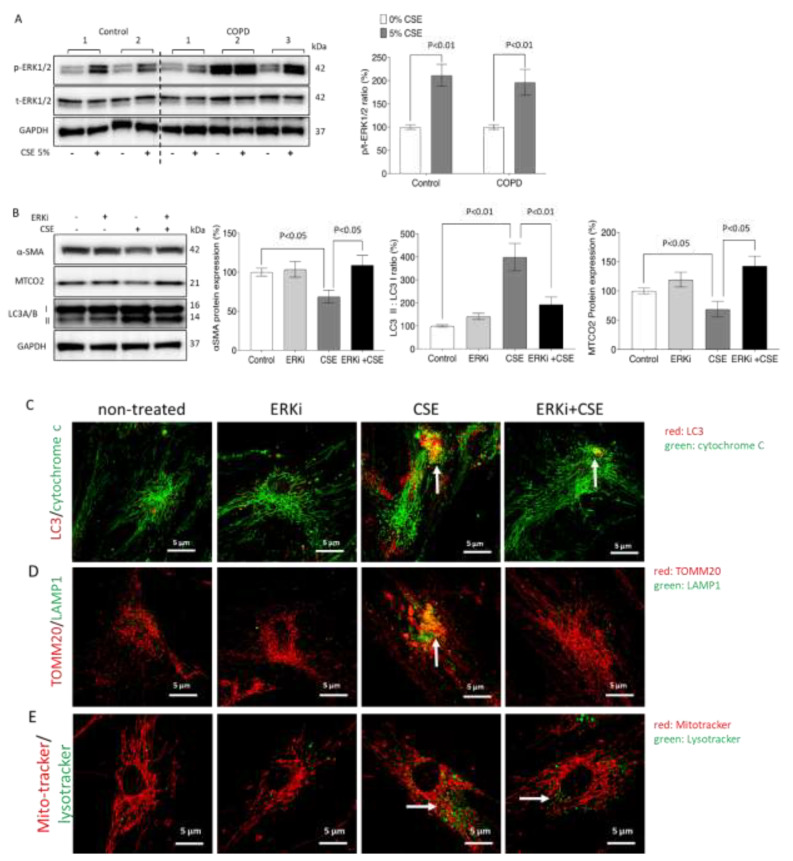
CSE activates ASMC remodeling, mitochondria activity, and mitophagy through ERK1/2. (**A**) Representative Western blots and image analysis of CSE-induced ERK1/2 phosphorylation. GAPDH served as a reference protein and *p* < 0.05 was considered significant. (**B**) Representative Western blots of α-SMA, MTCO2, and LC3A/B protein expression. GAPDH served as a reference protein and *p* < 0.05 was considered significant. (**C**–**E**) Representative confocal microscopy of mitochondria–LC3 co-localization (white arrow). (**D**) Mitochondria–lysosome co-localization (white arrow). (**E**) Live-cell imaging of mitochondria–lysosome co-localization (white arrow) after CSE-stimulation. (60×). Statistics were calculated by Mann–Whitney U-test.

In both groups of ASMCs, CSE treatment induced the phosphorylation of ERK1/2 MAPK by up to 200% when compared with untreated cells (Figure 6A). The concentration-dependent effect of CSE on ERK1/2 phosphorylation is shown in Figure S1C,D. By blocking the ERK1/2 MAPK phosphorylation 30 min before exposure to CSE, the stimulatory effect of CSE on the expression of the remodeling marker α-SMA, the mitochondria activity indicator MTCO2, and the autophagy marker LC3A/B were abrogated in the control ASMCs after CSE challenge (Figure 6B). 

The effect of ERK1/2 inhibition on CSE-induced mitophagy and mitochondria fragmentation was further assessed by immunofluorescence photography. As shown in Figure 6C, ERK1/2 inhibition significantly reduced the expression and co-localization of LC3 and cytochrome C. Color-dissected photographs of Figure 6C are provided in Figure S6. Similarly, inhibition of ERK1/2 abrogated the expression of LAMP1, while the expression of TOMM20 was not affected (Figure 6D). The reducing effect of ERK1/2 inhibition on LAMP1 is more visible in the color-dissected photographs shown in Figure S7. The inhibition of ERK1/2 also significantly reduced CSE-dependent lysosome activation (Figure 6E) Color-dissected photographs of Figure 6E are shown in Figure S8.

## 3. Discussion

In this study we provide further evidence that mitochondria structure and function are damaged on several levels in ASMCs of COPD patients. This damage is preserved even after several rounds of cell passaging, suggesting that it’s cause became endogenous. The phenotype of the mitochondria damage observed in COPD patient-derived cells was inducible in healthy ASMCs. In COPD cells, CSE treatment further increased the mitochondria damage, but due to pre-existing mitochondria pathologies, the effect was less prominent compared with healthy ASMCs. The data supports the idea that mitochondria regulation presents a therapeutic target that should be considered for COPD therapy [19,20,21,22].

Remodeling, especially of the small airway, is a pathology of COPD which is not affected by any of the available therapeutic strategies [20]. The role of ASMCs in COPD is controversially discussed, but it is implied that this cell type contributes to the local composition and turnover of the extracellular matrix (ECM). The function of ASMCs and their contribution to tissue remodeling is regulated by mitochondria [6]. In this study, we show that mitochondria homeostasis in ASMCs and tissue remodeling markers were modified by exposure to CSE. The effect of CSE on healthy ASMCs was very similar to the cellular pathologies that were detected in ASMCs of COPD patients. Therefore, this study provides evidence that cigarette smoke-induced imbalanced mitophagy of ASMCs is a contributing mechanism to lung remodeling in COPD. 

Aging of the lung seems to be accelerated by cigarette smoke and has become a target for developing new therapies for COPD [4,6,21]. Declining function and structure of mitochondria is one of the indicators for advanced aging. Oxidative stress had been suggested to cause mitochondrial dysfunction and increased the secretion of pro-inflammatory cytokines in an animal model and in ASMCs isolated from smokers and COPD patients [22]. However, the precise mechanism underlying this pathology in COPD was incomplete. In other studies, the data shows that CSE increases the fragmentation of mitochondria, which leads to increased synthesis of fibronectin by ASMCs which resembles the pathologies reported in COPD small airways [23,24]. 

Increased collagen type-I in serum was suggested as a marker for remodeling in COPD [25,26,27]. In this study, reduced collagen type-I expression was observed in ASMCs, which demonstrated a cell-type specific ECM production imbalance. In tissue sections of COPD patients, a reduced content of collagen type-I and an increased deposition of fibronectin in the area of the small airways had been reported [28]. Increased circulating fibronectin released by macrophages was suggested to indicate ongoing remodeling in COPD [29]. Other studies indicated that fibronectin genes and protein expression do not correlate in COPD [30]. In this study, CSE upregulated both fibronectin and decreased collagen type-I synthesis by ASMCs. In the context of COPD and airway-wall remodeling, it should be taken into consideration that both epithelial cells and ASMCs have their independent influences on ECM depositions, while several ECM components are also sensitive to glucocorticoids and long acting β2-agonists, which are the most frequently prescribed drugs [31,32] 

Functional healthy mitochondria are critical for cell function and survival. Mitochondria regulate physiological processes, such as differentiation and programmed cell death (PCD), and they react to unfolded protein response (UPR) and reactive oxygen species (ROS). Therefore, maintaining the balance between mitochondria synthesis and turnover guarantees stable energy production [33]. Mitophagy is an essential mechanism to maintain mitochondria homeostasis and is controlled by multiple level pathways, including PTEN-induced putative protein kinase 1 (PINK1)-Parkin dependent protein ubiquitination [34], p62-LC3, and autophagosome formation [35], as well as selective proteasome/lysosome induced degradation [36,37]. The above presented data show that CSE significantly upregulated LC3A in ASMCs. Thus, CSE modifies the metabolism-regulating mitophagy and autophagy towards degradation. In this context, in Alzheimer’s disease, impaired mitophagy caused progressive accumulation of amyloid-β and hyper-phosphorylated tau, leading to neuron degeneration [38]. The inhibition of mitophagy in cardio-myocytes or macrophages were associated with cardiomyopathy or atherosclerosis [39]. In addition, Akt1-mediated mitophagy contributed to apoptosis resistance of alveolar macrophages and was responsible for pulmonary fibrosis [40].

The data presented above show that through ERK1/2 MAPK signaling, CSE reduced MTCO2 and MTCO4. Both enzymes indicate mitochondrial mass but are also key regulators of oxidative phosphorylation and aerobic energy generation, therefore ATP synthesis [41]. Thus, their reduced expression might limit the cells metabolism and lead to degeneration. Furthermore, our data show that PGC-1α, which is a major regulator of mitochondria metabolisms and oxidative stress, was downregulated by CSE. Others reported that PGC-1α is a central regulator of mitochondrial activity and was linked to the pathogenesis of cellular senescence in other diseases [42]. 

The contribution of mitophagy to the pathogenesis of COPD has been implicated in several studies, but the outcomes were controversial. Cigarette smoke-induced accumulation of mitochondria damage caused COPD and involved the PINK-PRKN pathway [43]. In this context, it was suggested that augmenting the autophagy or mitophagy might be used as a novel treatment strategy for COPD, especially emphysema [44]. In contrast, cigarette smoke-induced excessive mitophagy was responsible for the progression of COPD via programmed cell death (PCD) in lung epithelium [45]. Importantly, the effect of mitophagy might be cell type-specific in COPD. A lack of PINK-PARK signaling caused dysfunctional mitophagy and programmed cell necroptosis in bronchial epithelial cells in COPD, while causing senescence and myo-fibroblast differentiation in type-II alveolar epithelial cells [46,47,48]. In bronchial epithelial cells, CSE activated MAPK15-ULK1 signaling thereby increasing mitophagy [49]. With regards to the cell-type specific effect of mitophagy, the sub-epithelial remodeling of ASMCs might present a key player in COPD-associated tissue remodeling, which must be further investigated. 

The early endosome antigen 1 (EEA1) is an important molecule and, together with Rab5, guides endosomal trafficking and membrane fusion [50]. Therefore, it could be used to identify an early endosomal compartment or vesicles, as well as to indicate early formation of autophagosomes. In the above-described experiments, CSE increased the expression of both EEA1 and lysosomal-associated membrane protein 1 (LAMP1). The latter is an additional indicator of phagosome formation [51], and its co-localization with aggregated damaged mitochondria components demonstrated by confocal microscopy indicates that CSE-induced mitochondria autophagy is linked to the lysosomal degradation pathway. Additionally, the live-cell imaging result from Mitotracker-Lysotracker co-localization further confirmed this observation.

This study provides evidence which shows that ASMCs isolated from COPD patients present with deterioration, such as loss of α-SMA expression, but increased fibronectin production, which might lead to the disease-associated acceleration of cell senescence [43]. Furthermore, the data shows upregulated expression of p62, Beclin-1, and active LC3 in the ASMCs of COPD patients and in healthy cells treated with CSE. All three proteins and mitophagy were sensitive to the inhibition of ERK1/2 phosphorylation, which is in line with similar findings by others [52,53]. Furthermore, MTCO2 and MTCO4 expression was significantly reduced after exposure to CSE, which was prevented by inhibiting ERK1/2. Oxidative stress enhanced autophagy, leading to stem-cell death via ERK1/2 signaling [52]. In hepatocytes, oxidative stress activated ERK1/2, leading to mitophagy [53]. 

Similarly, mitophagy was induced by ERK1/2 signaling after treatment of laryngeal cancer cells with hydrogen peroxide [54]. The contribution of ERK1/2 signaling to the pathogenesis of COPD, especially tissue remodeling, has been reported earlier in epithelial cells and myo-fibroblasts [55,56,57]. Other studies linked the activation of myo-fibroblasts and epithelial-mesenchymal transition in COPD to ERK1/2 signaling [58,59]. Although these results suggest that ERK1/2 signaling is a major stimulator of several COPD-specific pathologies, and its inhibition may present a therapeutic target, inhibiting ERK1/2 signaling will affect many other physiological processes needed to maintain tissue and organ regeneration [59]. Thus, it must be considered that the activation of ERK1/2 by cigarette smoke may present the body with an attempt to counteract tissue and cell deterioration. 

Recent investigations suggested that the transport and recycling of mitochondria between different cell types is a mechanism that occurs under certain conditions and helps to maintain the integrity and function of tissues and organs. It was suggested that mesenchymal stem cells actively transfer mitochondria to damaged cells and thereby rescue them [60]. Therefore, the transportation of mitochondria might present a novel therapeutic option for COPD, which could be achieved in a cell type-specific manner [61,62]. 

The limitations of this study include: (i) the small sample size, due to the difficulty to obtain tissues from healthy donors and COPD patients; (ii) the CSE used in this study only contained water-soluble substances, so the effect of insoluble particles such as PM2.5 was lost; and (iii) the lack of an animal model to prove that inhibition of mitophagy in ASMCs prevents lung deterioration.

In conclusion, control of mitophagy might present a novel strategy to reduce the pathogenesis in COPD.

## 4. Materials and Methods

### 4.1. Materials

Primary human airway smooth cells were purchased from Lonza Pharma (Basel, Switzerland) and Biotech Bioscience Solution (Köln, Germany). The available tissue donor’s clinical data is presented in Table 1.

The 1R6F research cigarettes were obtained from the Kentucky Tobacco Research and Development Center (University of Kentucky, Lexington, KY, USA). 

Antibodies for ERK1/2 and *p*-ERK1/2 (Thr202/Tyr204), beclin1, LC3A/B, p62, MTCO4, TOMM20, and GAPDH were from Cell Signaling Technology (Beverly, MA, USA), and LAMP1, MTCO2, PGC-1α, EEA1 Collagen1, α-SMA, from Abcam (Cambridge, UK), cytochrome C was from BD Bioscience (Allschwil, Switzerland), fibronectin and fluorescents dye DAPI, Alexa-488, Alexa-546, Mitotracker DeepRed, and Lysotracker Green were from ThermoFisher Scientific (Waltham, MA, USA). More details of the antibody and the concentrations used are summarized in Table 2 below.

### 4.2. CSE Preparation

1R6F research cigarettes (University of Kentucky, US) were used to prepare CSE as described earlier [49,63]. Briefly, CSE was prepared freshly, and a total of 350 mL cigarette smoke was collected by a syringe and bubbled through 10 mL RPMI 1640 medium. The crude CSE was adjusted pH to 7.4 and filtered through a 0.22 μm filter. This solution was defined as 100% CSE, and working concentrations were prepared by dilution with cell culture medium.

### 4.3. Cell Culture and Stimulation

Primary human ASMCs were expanded as previously described [64]. In detail, human ASMCs were grown in DMEM medium supplemented with 5% smooth muscle growth supplement (SMGM, S00725) from Thermofisher, in a humidified incubator under 5% CO_2_ at 37 °C. Cells were seeded in T25 flasks or 8-well chamber slides (Sarstedt, Switzerland) until 70% confluency to add in either CSE or the specific inhibitors. Cell protein lysates were collected in RIPA buffer for further analysis, and slides were either fixed with 4% PFA buffer for immunofluorescence staining or directly proceed to live-cell imaging analysis.

### 4.4. Intracellular Signaling Evaluation

The ERK1/2 MAPK specific inhibitor (ERK Activation Inhibitor Peptide I, Cell-Permeable, cat# 328000) from Sigma-Aldrich (Buchs, Switzerland) was dissolved in water at 1mM as stock solution. Cells were treated with the ERK inhibitor (2.5 μM) 30 min before CSE stimulation at the IC-50 concentration suggested by the distributor.

### 4.5. Western Blot

Primary human ASMCs were homogenized in RIPA lysis buffer containing protease inhibitors (Sigma-Aldrich), and the protein concentration was quantified by BCA assay (ThermoFisher Scientific). Denatured protein was subjected to sodium dodecyl sulfate-polyacrylamide gel electrophoresis and transferred onto a nitrocellulose membrane. After blocking unspecific binding, the membranes were incubated with specific target antibodies or loading controls at 4 °C overnight. Protein bands were visualized after incubation of membranes with species-specific secondary HRP-conjugated antibody by chemiluminescence substrate using an Azure C300 digital imaging system (Axonlab, Baden, Switzerland), as described earlier [14].

### 4.6. Mitochondria Damage and Mitophagy Detection

The ASMCs on chamber slides were fixed in 4% paraformaldehyde for 15 min, permeabilized with 0.1% Triton X-100 for 15 min, and washed with PBS (phosphate buffer saline) at room temperature. After blocking with 5% bovine serum albumin in PBST (PBS + 0.1% TWEEN20) for 30 minutes at room temperature. The cells were incubated with specific primary antibodies targeting cytochrome c or TOMM20 alone, or in combination with LC3A/B or LAMP1 overnight at 4 °C. The cells were washed with PBS and incubated with a secondary antibody (30 min, 37 °C). Subsequently, the cells were stained with DAPI (5 min, room temperature). After washing with PBS, the stained cells were viewed using an A1R confocal laser scanning microscope (Nikon, Amsterdam, Netherlands). In addition, ASMCs were also stained in live-cell imaging conditions with LysoTracker Green (Thermo Fisher, L7526, 75 nM) to visualize lysosomes, and MitoTracker Red (Thermo Fisher, M22426, 250 nM) to visualize mitochondria. The co-localization of lysosomes and mitochondria was observed by a spinning disc confocal microscope (Nikon A1R, Amsterdam, The Netherlands).

### 4.7. Statistics

All data are presented as mean ± standard deviation (SD) and were analyzed using Prism9 software. The null hypothesis was that none of the treatments affected mitochondrial function and mitophagy. The statistical analysis was performed by Student’s *t*-test for the comparison between control and COPD groups. The effects of CSE on ASMCs were analyzed using a paired *t*-test or a Mann–Whitney U test. *p* < 0.05 was considered as significant.

## Figures and Tables

**Table 1:** Characteristics of ASMC donors as provided by the distributors.

Cell Line Donor	Age	Gender	Smoking Status
Control 1	57	male	Non-smoker
Control 2	52	female	Non-smoker
Control 3	65	male	Non-smoker
COPD 1	49	female	Active smoker
COPD 2	48	female	Active smoker
COPD 3	49	male	Active smoker
COPD 4	73	female	Active smoker
COPD 5	66	male	Active smoker
COPD 6	55	male	Active smoker

**Table 2:** Antibody list.

Name	Company	Catalog Nr.	Origin	Dilution
p-ERK1/2 (Thr202/Tyr204)	Cell Signaling Technology (CST)	9101	Rabbit	1:2000
ERK1/2	CST	9102	Rabbit	1:2000
Beclin-1	CST	3495	Rabbit	1:1000
LC3A/B	CST	12741	Rabbit	1:2000
MTCO4	CST	4850	Rabbit	1:2000
P62	R&D Systems	MAB8028	Mouse	1:1000
TOMM20	NOVUSBIO	MBP2-67501	Rabbit	1:500
Cytochrome c	BD Bioscience	556432	Mouse	1:1000
MTCO2	Abcam	Ab79393	Rabbit	1:1000
PGC-1α	Abcam	ab54481	Rabbit	1:500
GAPDH	Abcam	ab128915	Rabbit	1:5000
LAMP-1	Abcam	ab25630	Mouse	1:500
EEA1	Abcam	ab70521	Mouse	1:500
Collagen I	Abcam	ab34710	Rabbit	1:1000
α-SMA	Abcam	ab124964	Rabbit	1:5000
Fibronectin	Thermo-Fisher	MA5-11981	Mouse	1:500
Anti-Ms-Alexa-488	Thermo-Fisher	A21121	Goat	1:500
Anti-Rab-Alexa-546	Thermo-Fisher	A11010	Goat	1:500
Anti-mouse-HRP	Sigma-Aldrich	A9917	Goat	1:2000
Anti-rabbit-HRP	Sigma-Aldrich	A9169	Goat	1:2000

## Data Availability

Original data can be requested from the first author.

## Supplementary Materials

The supporting information can be downloaded at: https://www.mdpi.com/article/10.3390/ijms232213987/s1.

## References

[1] Huynh D.T.N., Heo K.S. (2021). Role of mitochondrial dynamics and mitophagy of vascular smooth muscle cell proliferation and migration in progression of atherosclerosis. Arch. Pharm. Res..

[2] Pham A.H., McCaffery J.M., Chan D.C. (2021). Mouse lines with photo-activatable mitochondria to study mitochondrial dynamics. Genesis.

[3] Levine B., Kroemer G. (2019). Biological functions of autophagy genes: A disease perspective. Cell.

[4] Chen G., Kroemer G., Kepp O. (2020). Mitophagy: An Emerging Role in Aging and Age-Associated Diseases. Front. Cell Dev. Biol..

[5] Sharma A., Ahmad S., Ahmad T., Ali S., Syed M.A. (2021). Mitochondrial dynamics and mitophagy in lung disorders. Life Sci..

[6] Barnes P.J., Baker J., Donnelly L.E. (2022). Autophagy in asthma and chronic obstructive pulmonary disease. Clin. Sci. Lond..

[7] Jones R.L., Noble P.B., Elliot J.G., James A.L. (2016). Airway remodelling in COPD: It’s not asthma!. Respirology.

[8] Esteves P., Blanc L., Celle A., Dupin I., Maurat E., Amoedo N., Cardouat G., Ousova O., Gales L., Bellvert F. (2021). Crucial role of fatty acid oxidation in asthmatic bronchial smooth muscle remodelling. Eur. Respir. J..

[9] Dasgupta D., Delmotte P., Sieck G.C. (2020). Inflammation-Induced Protein Unfolding in Airway Smooth Muscle Triggers a Homeostatic Response in Mitochondria. Int. J. Mol. Sci..

[10] Cipollina C., Bruno A., Fasola S., Cristaldi M., Patella B., Inguanta R., Vilasi A., Aiello G., La Grutta S., Torino C. (2022). Cellular and Molecular Signatures of Oxidative Stress in Bronchial Epithelial Cell Models Injured by Cigarette Smoke Extract. Int. J. Mol. Sci..

[11] Pace E., Di Vincenzo S., Di Salvo E., Genovese S., Dino P., Sangiorgi C., Ferraro M., Gangemi S. (2019). MiR-21 upregulation increases IL-8 expression and tumorigenesis program in airway epithelial cells exposed to cigarette smoke. J. Cell Physiol..

[12] Hara H., Araya J., Ito S., Kobayashi K., Takasaka N., Yoshii Y., Wakui H., Kojima J., Shimizu K., Numata T. (2013). Mitochondrial fragmentation in cigarette smoke-induced bronchial epithelial cell senescence. Am. J. Physiol. Lung Cell Mol. Physiol..

[13] Ito S., Araya J., Kurita Y., Kobayashi K., Takasaka N., Yoshida M., Hara H., Minagawa S., Wakui H., Fujii S. (2015). PARK2-mediated mitophagy is involved in regulation of HBEC senescence in COPD pathogenesis. Autophagy.

[14] Fang L., Wang X., Sun Q., Papakonstantinou E., S’ng C., Tamm M., Stolz D., Roth M. (2019). IgE Downregulates PTEN through MicroRNA-21-5p and Stimulates Airway Smooth Muscle Cell Remodeling. Int. J. Mol. Sci..

[15] Mc Lelland G.L., Goiran T., Yi W., Dorval G., Chen C.X., Lauinger N.D., Krahn A.I., Valimehr S., Rakovic A., Rouiller I. (2018). Mfn2 ubiquitination by PINK1/parkin gates the p97-dependent release of ER from mitochondria to drive mitophagy. Elife.

[16] Lau J.Y., Oliver B.G., Moir L.M., Black J.L., Burgess J.K. (2010). Differential expression of peroxisome proliferator activated receptor gamma and cyclin D1 does not affect proliferation of asthma- and non-asthma-derived airway smooth muscle cells. Respirology.

[17] Ward J.E., Gould H., Harris T., Bonacci J.V., Stewart A.G. (2004). PPAR gamma ligands, 15-deoxy-delta12,14-prostaglandin J2 and rosiglitazone regulate human cultured airway smooth muscle proliferation through different mechanisms. Br. J. Pharmacol..

[18] Su Y., Han W., Kovacs-Kasa A., Verin A.D., Kovacs L. (2021). HDAC6 Activates ERK in Airway and Pulmonary Vascular Remodeling of Chronic Obstructive Pulmonary Disease. Am. J. Respir. Cell Mol. Biol..

[19] Aravamudan B., Thompson M., Sieck G.C., Vassallo R., Pabelick C.M., Prakash Y.S. (2017). Functional Effects of Cigarette Smoke-Induced Changes in Airway Smooth Muscle Mitochondrial Morphology. J. Cell Physiol..

[20] Lareau S.C., Fahy B., Meek P., Wang A. (2019). Chronic Obstructive Pulmonary Disease (COPD). Am. J. Respir. Crit. Care Med..

[21] Easter M., Bollenbecker S., Barnes J.W., Krick S. (2020). Targeting Aging Pathways in Chronic Obstructive Pulmonary Disease. Int. J. Mol. Sci..

[22] Wiegman C.H., Michaeloudes C., Haji G., Narang P., Clarke C.J., Russell K.E., Bao W., Pavlidis S., Barnes P.J., Kanerva J. (2015). Oxidative stress-induced mitochondrial dysfunction drives inflammation and airway smooth muscle remodeling in patients with chronic obstructive pulmonary disease. J. Allergy Clin. Immunol..

[23] Harju T., Kinnula V.L., Pääkkö P., Salmenkivi K., Risteli J., Kaarteenaho R. (2010). Variability in the precursor proteins of collagen I and III in different stages of COPD. Respir. Res..

[24] Tjin G., Xu P., Kable S.H., Kable E.P., Burgess J.K. (2014). Quantification of collagen I in airway tissues using second harmonic generation. J. Biomed. Opt..

[25] Stolz D., Leeming D.J., Kristensen J.H.E., Karsdal M.A., Boersma W., Louis R., Milenkovic B., Kostikas K., Blasi F., Aerts J. (2017). Systemic Biomarkers of Collagen and Elastin Turnover Are Associated With Clinically Relevant Outcomes in COPD. Chest.

[26] Zeng Y.Y., Hu W.P., Zuo Y.H., Wang X.R., Zhang J. (2019). Altered serum levels of type I collagen turnover indicators accompanied by IL-6 and IL-8 release in stable COPD. Int. J. Chron. Obstruct Pulmon. Dis..

[27] Bihlet A.R., Karsdal M.A., Sand J.M., Leeming D.J., Roberts M., White W., Bowler R. (2017). Biomarkers of extracellular matrix turnover are associated with emphysema and eosinophilic-bronchitis in COPD. Respir. Res..

[28] Annoni R., Lanças T., Yukimatsu Tanigawa R., de Medeiros Matsushita M., de Morais Fernezlian S., Bruno A., Fernando Ferraz da Silva L., Roughley P.J., Battaglia S., Dolhnikoff M. (2012). Extracellular matrix composition in COPD. Eur. Respir. J..

[29] Song W.D., Zhang A.C., Pang Y.Y., Liu L.H., Zhao J.Y., Deng S.H., Zhang S.Y. (1995). Fibronectin and hyaluronan in bronchoalveolar lavage fluid from young patients with chronic obstructive pulmonary diseases. Respiration.

[30] Muñoz-Esquerre M., Huertas D., Escobar I., López-Sánchez M., Penín R., Peinado V., Barberà J.A., Molina-Molina M., Manresa F., Dorca J. (2015). Gene and Protein Expression of Fibronectin and Tenascin-C in Lung Samples from COPD Patients. Lung.

[31] Degen M., Goulet S., Ferralli J., Roth M., Tamm M., Chiquet-Ehrismann R. (2009). Opposite effect of fluticasone and salmeterol on fibronectin and tenascin-C expression in primary human lung fibroblasts. Clin. Exp. Allergy.

[32] Goulet S., Bihl M.P., Gambazzi F., Tamm M., Roth M. (2007). Opposite effect of corticosteroids and long-acting beta(2)-agonists on serum- and TGF-beta(1)-induced extracellular matrix deposition by primary human lung fibroblasts. J. Cell Physiol..

[33] Pickles S., Vigié P., Youle R.J. (2018). Mitophagy and Quality Control Mechanisms in Mitochondrial Maintenance. Curr. Biol..

[34] Ashrafi G., Schwarz T.L. (2013). The pathways of mitophagy for quality control and clearance of mitochondria. Cell Death Differ..

[35] Ye J., Zheng M. (2021). Autophagosome Trafficking. Adv. Exp. Med. Biol..

[36] Nguyen T.N., Padman B.S., Usher J., Oorschot V., Ramm G., Lazarou M. (2016). Atg8 family LC3/GABARAP proteins are crucial for autophagosome-lysosome fusion but not autophagosome formation during PINK1/Parkin mitophagy and starvation. J. Cell Biol..

[37] Lamark T., Svenning S., Johansen T. (2017). Regulation of selective autophagy: The p62/SQSTM1 paradigm. Essays Biochem..

[38] Pradeepkiran J.A., Reddy P.H. (2020). Defective mitophagy in Alzheimer’s disease. Ageing Res. Rev..

[39] Bravo-San Pedro J.M., Kroemer G., Galluzzi L. (2017). Autophagy and Mitophagy in Cardiovascular Disease. Circ. Res..

[40] Larson-Casey J.L., Deshane J.S., Ryan A.J., Thannickal V.J., Carter A.B. (2016). Macrophage Akt1 Kinase-Mediated Mitophagy Modulates Apoptosis Resistance and Pulmonary Fibrosis. Immunity..

[41] Timón-Gómez A., Nývltová E., Abriata L.A., Vila A.J., Hosler J., Barrientos A. (2018). Mitochondrial cytochrome c oxidase biogenesis: Recent developments. Semin. Cell Dev. Biol..

[42] Austin S., St-Pierre J. (2012). PGC1α and mitochondrial metabolism--emerging concepts and relevance in ageing and neurodegenerative disorders. J. Cell Sci..

[43] Araya J., Tsubouchi K., Sato N., Ito S., Minagawa S., Hara H., Hosaka Y., Ichikawa A., Saito N., Kadota T. (2019). PRKN-regulated mitophagy and cellular senescence during COPD pathogenesis. Autophagy.

[44] Bodas M., Vij N. (2017). Augmenting autophagy for prognosis based intervention of COPD-pathophysiology. Respir. Res..

[45] Mizumura K., Cloonan S.M., Nakahira K., Bhashyam A.R., Cervo M., Kitada T., Glass K., Owen C.A., Mahmood A., Washko G.R. (2014). Mitophagy-dependent necroptosis contributes to the pathogenesis of COPD. J. Clin. Investig..

[46] Tsubouchi K., Araya J., Kuwano K. (2018). PINK1-PARK2-mediated mitophagy in COPD and IPF pathogeneses. Inflamm. Regen..

[47] Ryter S.W., Rosas I.O., Owen C.A., Martinez F.J., Choi M.E., Lee C.G., Elias J.A., Choi A.M.K. (2018). Mitochondrial Dysfunction as a Pathogenic Mediator of Chronic Obstructive Pulmonary Disease and Idiopathic Pulmonary Fibrosis. Ann. Am. Thorac. Soc..

[48] Roque W., Cuevas-Mora K., Romero F. (2020). Mitochondrial Quality Control in Age-Related Pulmonary Fibrosis. Int. J. Mol. Sci..

[49] Zhang M., Fang L., Zhou L., Molino A., Valentino M.R., Yang S., Zhang J., Li Y., Roth M. (2021). MAPK15-ULK1 signaling regulates mitophagy of airway epithelial cell in chronic obstructive pulmonary disease. Free Radic. Biol. Med..

[50] Mills I.G., Jones A.T., Clague M.J. (1999). Regulation of endosome fusion. Mol. Membr. Biol..

[51] Eskelinen E.L. (2006). Roles of LAMP-1 and LAMP-2 in lysosome biogenesis and autophagy. Mol. Aspects Med..

[52] Prakash R., Fauzia E., Siddiqui A.J., Yadav S.K., Kumari N., Singhai A., Khan M.A., Janowski M., Bhutia S.K., Raza S.S. (2021). Oxidative Stress Enhances Autophagy-Mediated Death Of Stem Cells Through Erk1/2 Signaling Pathway—Implications For Neurotransplantations. Stem. Cell. Rev. Rep..

[53] Chen J., Wang D., Zong Y., Yang X. (2021). DHA Protects Hepatocytes from Oxidative Injury through GPR120/ERK-Mediated Mitophagy. Int. J. Mol. Sci..

[54] Hui L., Wu H., Wang T.W., Yang N., Guo X., Jang X.J. (2019). Hydrogen peroxide-induced mitophagy contributes to laryngeal cancer cells survival via the upregulation of FUNDC1. Clin. Transl. Oncol..

[55] Vij N., Chandramani-Shivalingappa P., Van Westphal C., Hole R., Bodas M. (2018). Cigarette smoke-induced autophagy impairment accelerates lung aging, COPD-emphysema exacerbations and pathogenesis. Am. J. Physiol. Cell Physiol..

[56] Tao Y., Sun Y., Wu B., Xu D., Yang J., Gu L., Du C. (2021). Overexpression of FOXA2 attenuates cigarette smoke-induced cellular senescence and lung inflammation through inhibition of the p38 and Erk1/2 MAPK pathways. Int. Immunopharmacol..

[57] Kim H., Liu X., Kohyama T., Kobayashi T., Conner H., Abe S., Fang Q., Wen F.Q., Rennard S.I. (2004). Cigarette smoke stimulates MMP-1 production by human lung fibroblasts through the ERK1/2 pathway. COPD.

[58] Liu Y.N., Guan Y., Shen J., Jia Y.L., Zhou J.C., Sun Y., Jiang J.X., Shen H.J., Shu Q., Xie Q.M. (2020). Shp2 positively regulates cigarette smoke-induced epithelial mesenchymal transition by mediating MMP-9 production. Respir. Res..

[59] Wen X., Jiao L., Tan H. (2022). MAPK/ERK Pathway as a Central Regulator in Vertebrate Organ Regeneration. Int. J. Mol. Sci..

[60] Mahrouf-Yorgov M., Augeul L., Da Silva C.C., Jourdan M., Rigolet M., Manin S., Ferrera R., Ovize M., Henry A., Guguin A. (2017). Mesenchymal stem cells sense mitochondria released from damaged cells as danger signals to activate their rescue properties. Cell Death Differ..

[61] Michaeloudes C., Li X., Mak J.C.W., Bhavsar P.K. (2021). Study of Mesenchymal Stem Cell-Mediated Mitochondrial Transfer in In Vitro Models of Oxidant-Mediated Airway Epithelial and Smooth Muscle Cell Injury. Methods Mol. Biol..

[62] Frankenberg Garcia J., Rogers A.V., Mak J.C.W., Halayko A.J., Hui C.K., Xu B., Chung K.F., Rodriguez T., Michaeloudes C., Bhavsar P.K. (2022). Mitochondrial Transfer Regulates Bioenergetics in Healthy and COPD Airway Smooth Muscle. Am. J. Respir. Cell Mol. Biol..

[63] Vayssier-Taussat M., Camilli T., Aron Y., Meplan C., Hainaut P., Polla B.S., Weksler B. (2021). Effects of tobacco smoke and benzo[a]pyrene on human endothelial cell and monocyte stress responses. Am. J. Physiol. Heart Circ. Physiol..

[64] Johnson P.R., Roth M., Tamm M., Hughes M., Ge Q., King G., Burgess J.K., Black J.L. (2001). Airway smooth muscle cell proliferation is increased in asthma. Am. J. Respir. Crit. Care Med..

