# Supplementary material for: Airway Smooth Muscle Cell Mitochondria Damage and Mitophagy in COPD via ERK1/2 MAPK
Source: Int J Mol Sci. 2022 Nov 12;23(22):13987. doi: 10.3390/ijms232213987 (PMC9694999; doi:10.3390/ijms232213987)
Supplement: Supplementary file 1 [file ijms-23-13987-s001.zip › ijms-1947210-supplementary.pdf]

Figure S1

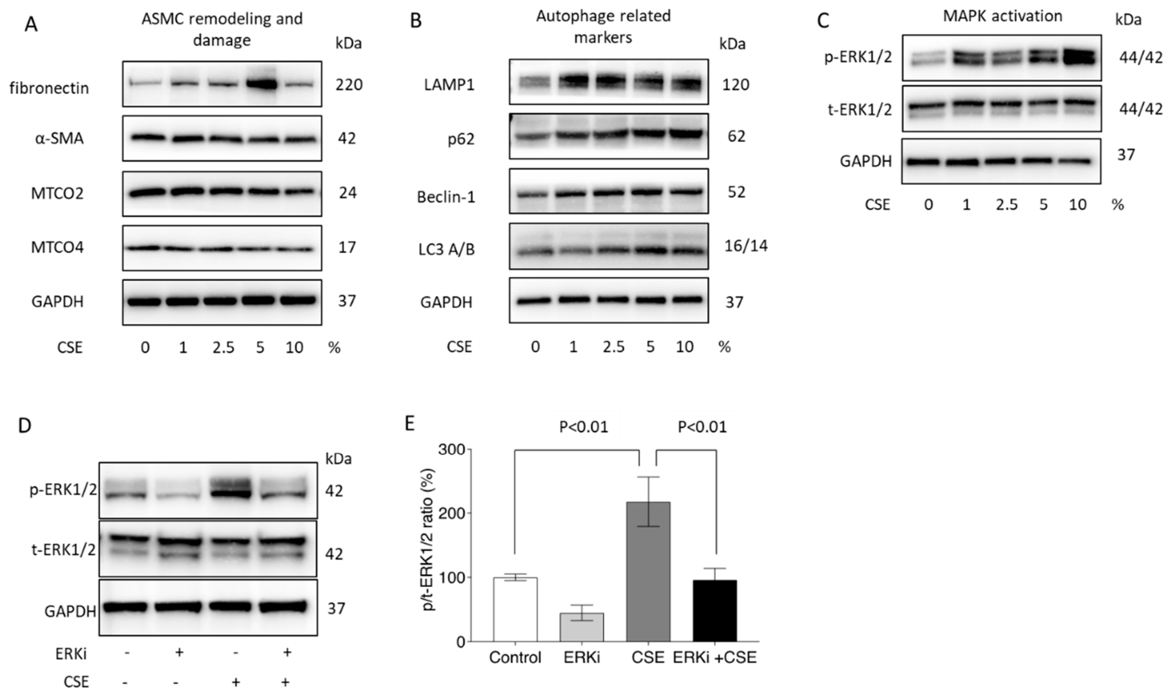

Figure S1: Concentration dependency of CSE-induced remodeling. A, Representative Western-blots of the concentration-dependent effect of CSE on control ASMC fibronectin, αSMA, MTCO2, MTCO4 protein expressions. **B,** Representative Western-blots of the dose-dependent effect of CSE on control ASMC LC3A/B, Beclin-1, p62, and LAMP1 protein expressions. **C**, Representative Western-blots of the dose-dependent effect of CSE on control ASMC ERK1/2 protein phosphorylation. **D & E** The role of ERK1/2 in CSE-induced phosphorylation in the presence and absence of ERK inhibitor (ERKi); $p<0.05$ was considered significant.

Figure S2

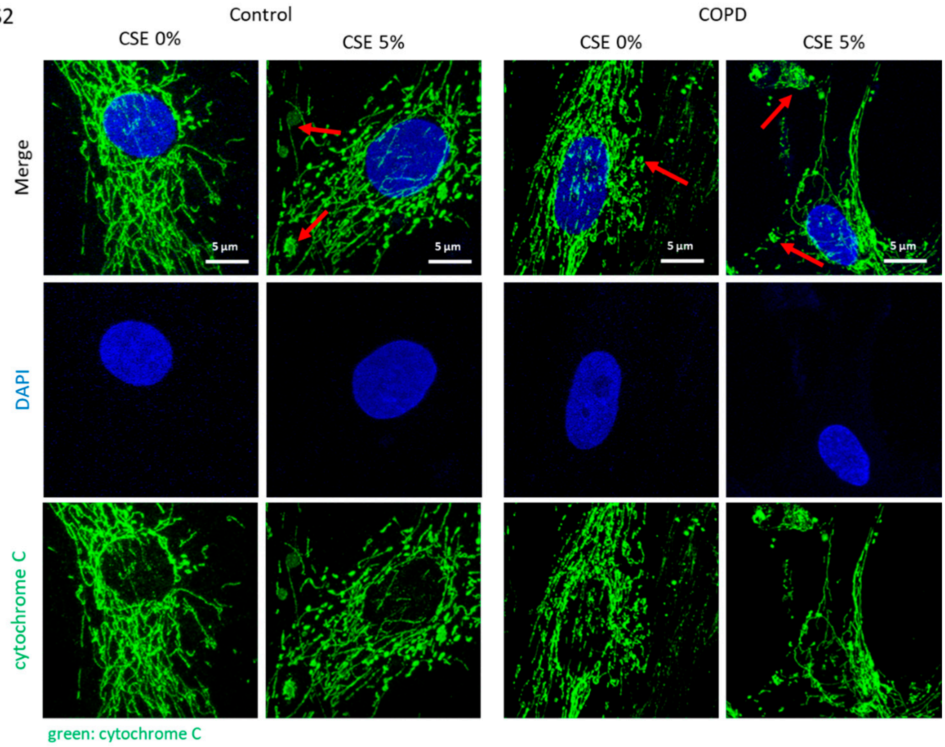

Figure S2: Color dissection of CES-induced mitochondria fragmentation. Representative color dissected photographs of Figure 3C (60X, green: cytochrome C, blue: DAPI, red arrow indicates fragmented mitochondria determined by cytochrome C).

Figure S3

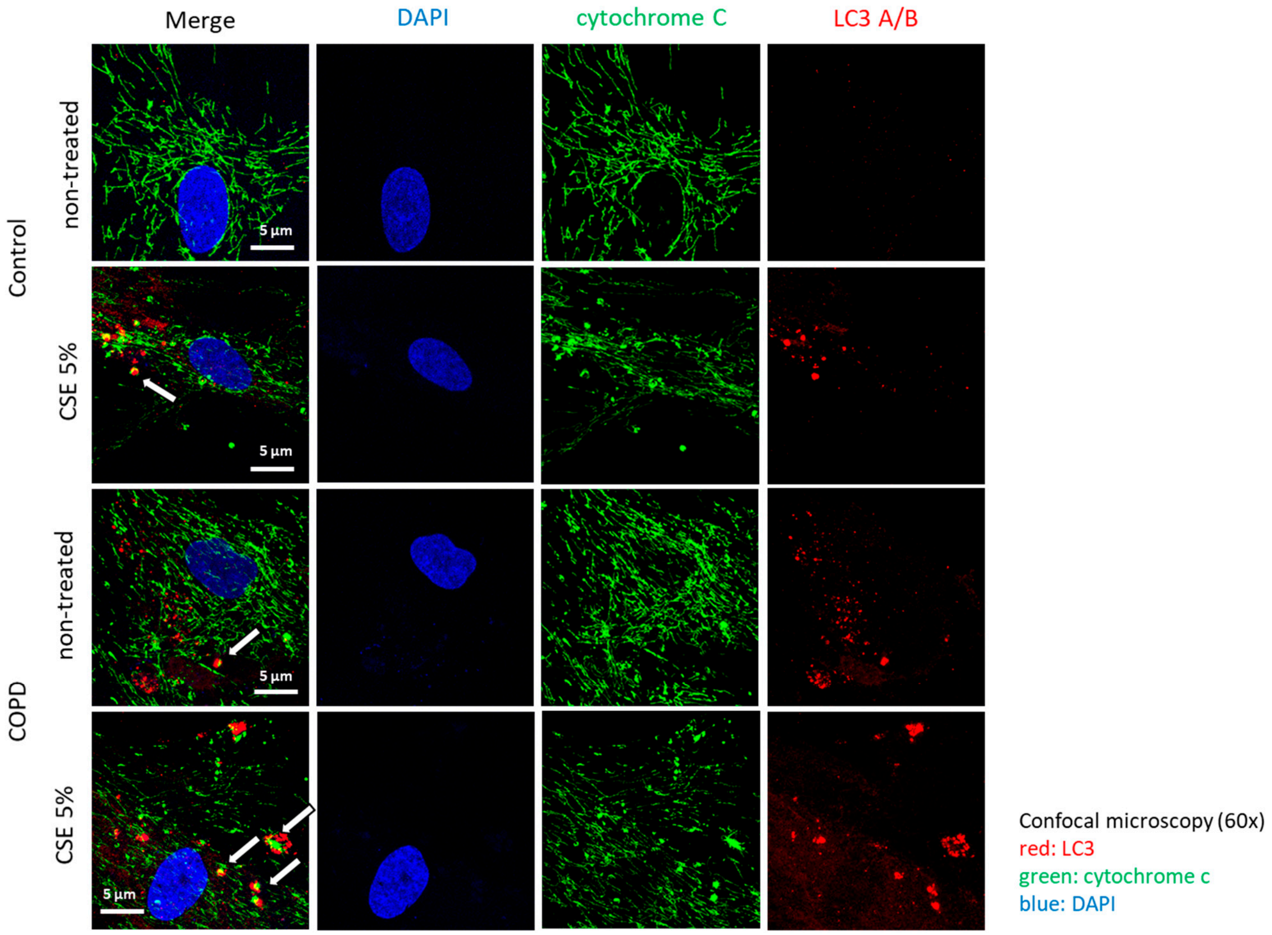

Figure S3: Color dissection of CSE-induced mitochondria and LC3A7B co-localization. Representative color dissected photographs of Figure 4B (60X, green: cytochrome C, red: LC3A/B, blue: DAPI, white arrow indicates co-localization of mitochondria and LC3A/B).

Figure S4

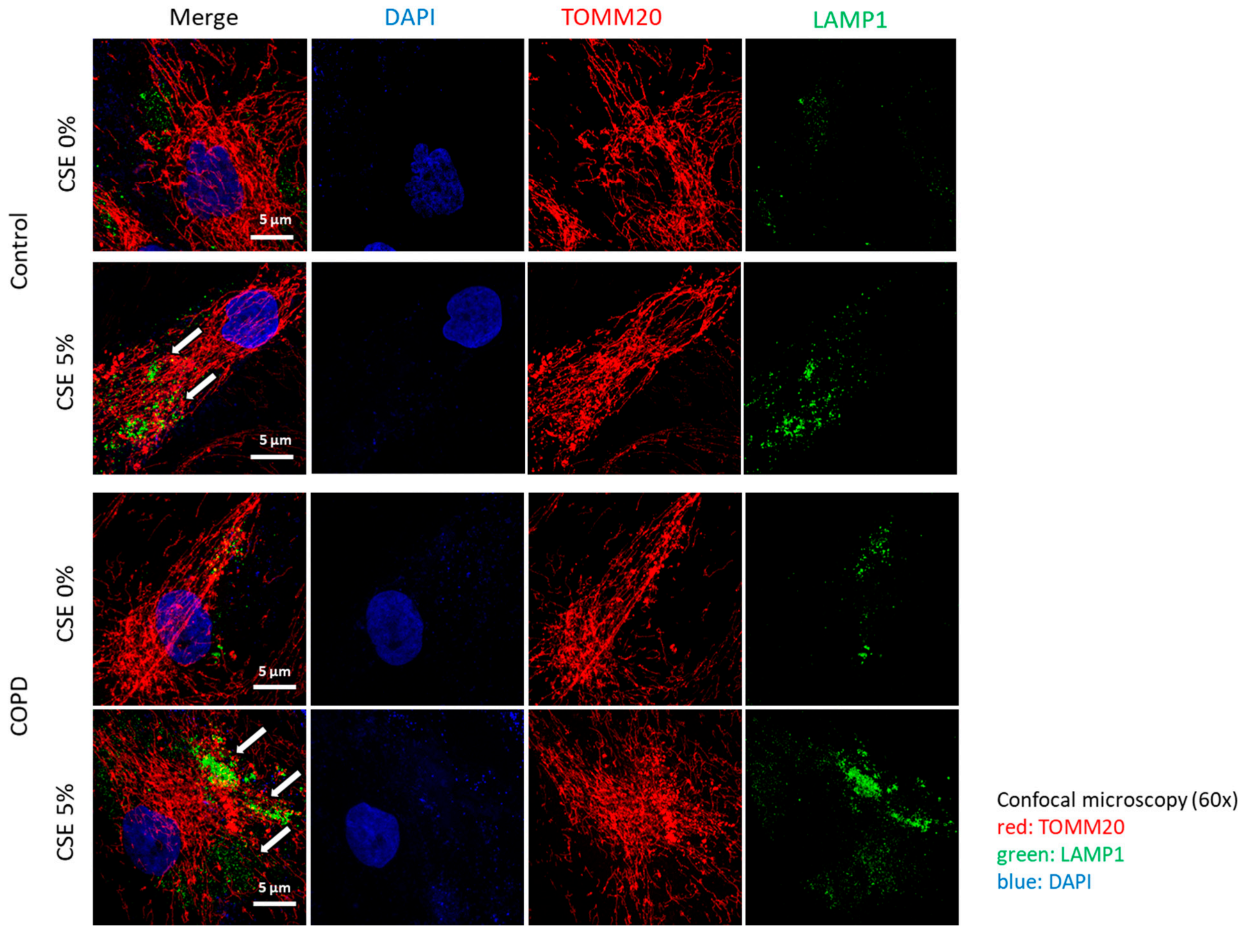

Figure S4: Color dissection of CSE-induced lysosome activity. Representative color dissected photographs of Figure 5B (60X, red: TOMM20, green: LAMP1, blue: DAPI, white arrow indicates co-localization of mitochondria and lysosome).

Figure S5

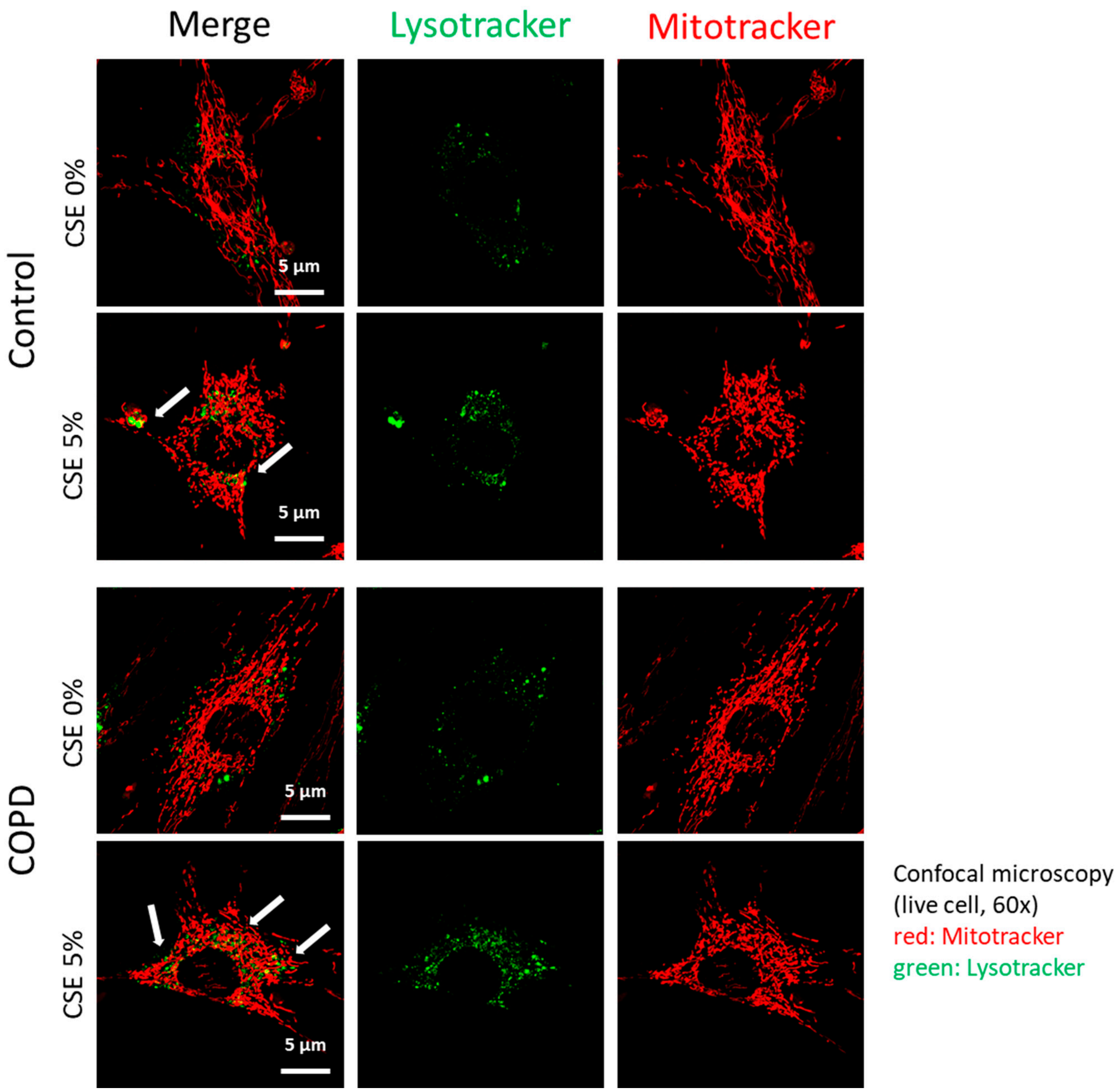

Figure S5: Color dissection of CSE-induced Mito- and Lysotracker co-localisation. Representative color dissected photographs of Figure 5C (60X, red: Mitotracker, green: Lysotracker, white arrow indicates co-localization of mitochondria and lysosome).

Figure S6

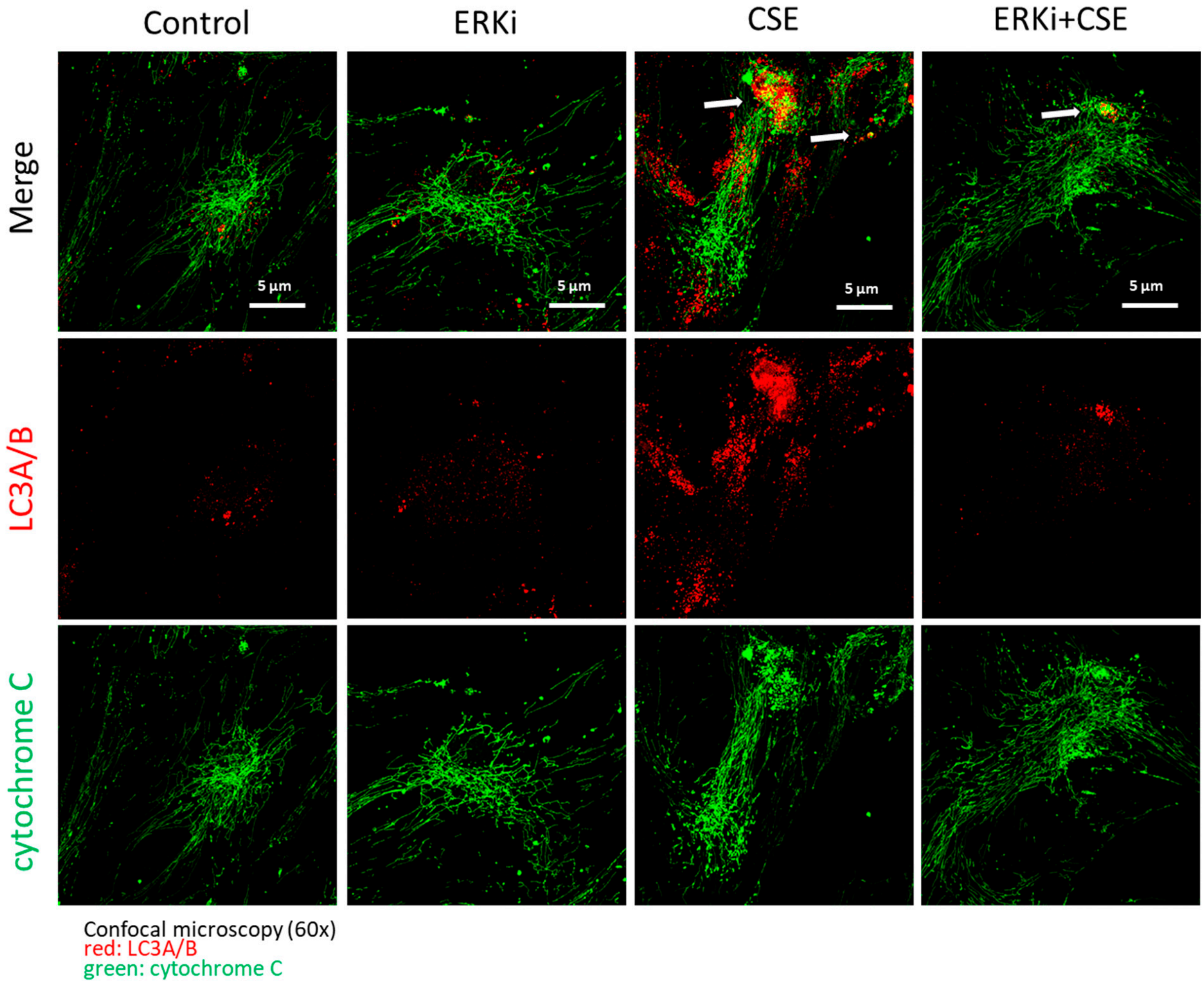

Figure S6: Color dissection of ERK1/2 inhibition on CSE-induced cytochrome C and LC3A/B co-localization. Representative color dissected photographs of Figure 6C (60X, green: cytochrome C, red: LC3A/B, white arrow indicates co-localization of cytochrome C and LC3A/B).

Figure S7

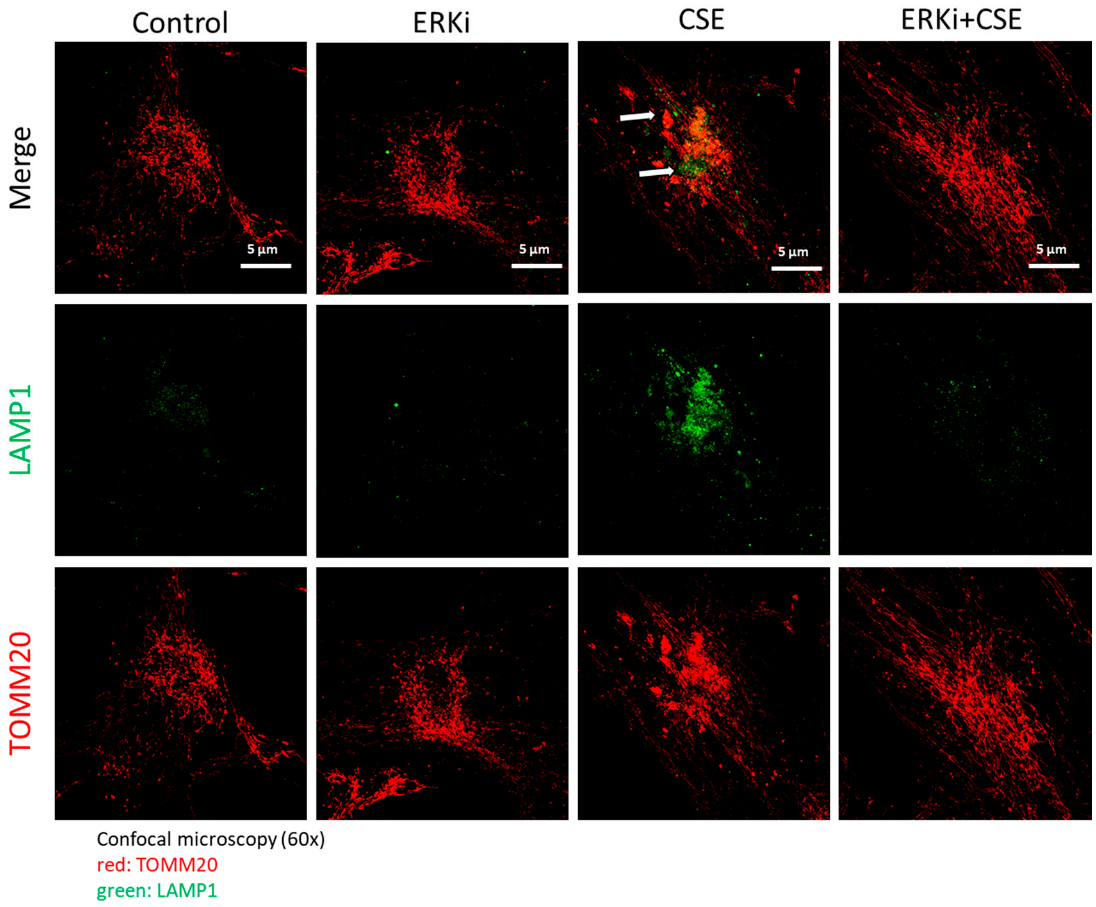

Figure S7: Color dissection of ERK1/2 inhibition on CSE-induced lysosome activity. Representative color dissected photographs of Figure 6D (60X, red: TOMM20, green: LAMP1, white arrow indicates co-localization of mitochondria and lysosome).

Figure S8

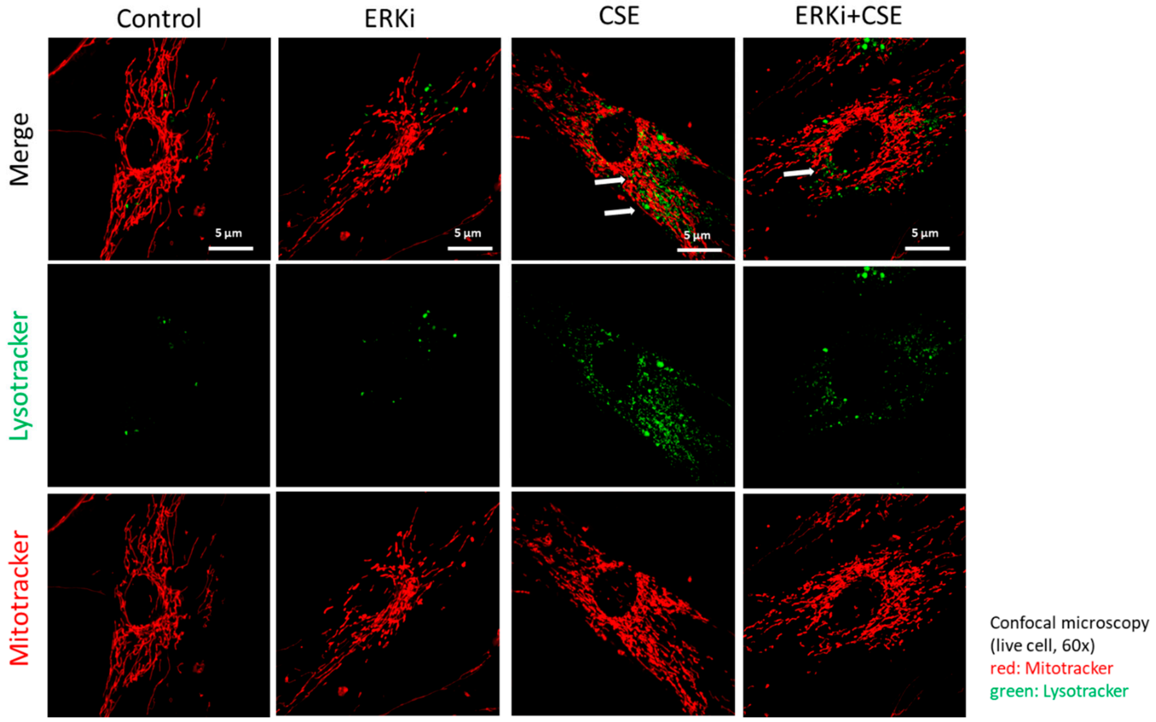

Figure S8: Color dissection of ERK1/2 inhibition on CSE-induced Mio- and Lysotracker activity. Representative color dissected photographs of Figure 6E (60X, red: Mitotracker, green: Lysotracker, white arrow indicates co-localization of mitochondria and lysosome).
